# Supplementary material for: Could Greater Time Spent Displaying Waking Inactivity in the Home Environment Be a Marker for a Depression-Like State in the Domestic Dog?
Source: Animals (Basel). 2019 Jul 5;9(7):420. doi: 10.3390/ani9070420 (PMC6680786; doi:10.3390/ani9070420)
Supplement: Supplementary file 1 [file animals-09-00420-s001.pdf]

Supplementary Material

Could greater time spent being awake but motionless in the home environment be a marker for a depression-like state in the domestic dog?

Naomi D. Harvey [1*], Alexandra Moesta[2], Sarah Kappel[3], Chanakarn Wongsaengchan[1,3], Hannah Harris[1,3], Peter Craigon[1,3], Carole Fureix [3]

[1] School of Veterinary Medicine and Science, University of Nottingham, Sutton Bonington LE12 5RD, UK
[2] WALTHAM Centre for Pet Nutrition, Waltham-on-the-Wolds, Melton Mowbray, LE14 4 RT, UK; current address: Royal Canin Research Center, 30470 Aimargues, France.
[3] School of Biological and Marine Sciences, University of Plymouth, Plymouth PL4 8AA, UK; part of this work was also conducted at the University of Bristol, Langford, BS40 5DU
* Correspondence: Naomi.Harvey@nottingham.ac.uk;

Pilot Study

Methods

This study was conducted in two parts: a pilot phase, where preliminary data were collected, analysed and used to conduct a sample size estimation (described here) and qualitative behaviour analyses (see main paper), and a full study on an independent sample to meet sample size requirement (see main paper). Three pilot shelters were recruited in total: *shelter 1* (in which observations were carried in March, June and September 2016), *shelter B* (August 2016) and *shelter C* (October 2016). Shelter A was where the methods used in this study were refined and planned before data collection commenced. A total of 18 dogs had complete datasets. These 18 dogs formed the basis of this pilot study (distributed across the shelters 1, 2 and 3 as 10, 3 and 5 dogs, respectively) and were aged 1-9 years (average 2.9 ± 2.06 years), with a 50:50 ratio of crossbreds to purebreds and males to females.

Dogs housing conditions, home-pen observations (quantifying time spent being awake but motionless) and Kong™ anhedonia test are similar to those described in the Methods section of the main paper, with the exception of the Kong™ variables (that we refined following the pilot study). Kong™ measures for the pilot study were: the total time the dog spent interacting with the KongTM (defined as: paw or muzzle in contact with, or sniffing the KongTM, excluding time stood chewing not in contact) (*Kong™ Time*); the time to interact with KongTM from first placement on floor (*Kong™ Latency*); the number of times dog returned to contact Kong after moving >2 paces away (*Kong™ Returns*). Data regarding the percentage of the food mix eaten by the end of the 30 minutes test was available for the main study dogs only.

Although ABM was not normally distributed, both univariate and multivariate model residuals were visually examined and considered to be acceptable, so no transformations were applied.

Interval sample selection

In order to be time efficient, but not loose accuracy of the data, it was necessary to identify the maximum sample interval we could use that would produce a representative activity budget. Shorter sample intervals form more accurate representations of behaviour but are less time

and cost effective than longer intervals (Hämäläinen, Ruuska, Kokkonen, Orkola, & Mononen, 2016).

For the first seven dogs from Shelter A scans were taken every 30 seconds, over the entire 6hr recording period. Inevitably, dogs were not always visible for the entire 6hrs of footage, with some dogs being taken out for walks during a recording period or kennels being cleaned. For this reason, the number of visible scans was summed for each period per dog, and the minimum number of visible scans for each dog identified. In this way it was confirmed that each of the initial 7 dogs had at least 160 visible scans (80 minutes) per 2hr period and 4 hours (480 scans) in total; this was the maximum number of visible scans that could be met for every dog. Therefore, while some dogs had more than 160 scans visible, we limited all dogs to 160 visible scans to ensure all dogs had the same number of visible scans and that all dogs could be compared in a comparative manner. For a dog with more than 160 visible scans in any period, 160 visible scans were identified, iteratively, from the centre of the largest consecutive block of visible scans.

An activity budget was calculated for each dog comprising of the proportion of scans seen exhibiting each point event from the ethogram. This was done iteratively for all 7 dogs for the original 30-second interval scans, then utilising every 2nd scan (representing 1-minute intervals), every 3rd scan (representing 1.5-minute intervals) and every 4th scan (representing 2-minute intervals), creating 4 different datasets.

The mean proportion of scans spent in each behavioural state was calculated for each dataset, followed by the difference in the mean between the 30-second reference data and each of the 2nd, 3rd and 4th scan data (the Error Proportion; EP). If the EP for the larger interval datasets for any behavioural state was less than 10% different from of the 30-second mean estimate then it was considered to have retained accuracy (Hämäläinen et al., 2016). In this way, the longest interval that produced mean behavioural estimates most similar to the 30-second reference sample, for the greatest number of behavioural states, was selected for subsequent video analysis.

Results

Time budget estimates remained very similar for all scan intervals between 30-seconds and 2 minutes. However, accuracy at longer intervals decreased for behaviour with the shortest durations. For 1-minute intervals, the only behavioural estimates that changed by more than 10% were those that were exhibited for less than 1% of the time, and at 1.5-minute intervals for those that were exhibited for less than 2% of the time (Table 1). At 1 and 1.5-minute intervals, all estimates for the behaviour essential to this study (doing nothing) were all within a 10% margin of the 30-second interval estimates. Two-minute intervals produced a greater difference in estimates and a marked loss of accuracy for behaviour including lying down head or ears mobile, sleeping and walking. For these reasons, the 1.5-minute interval was deemed to produce acceptably accurate time budget estimates and was utilised for all subsequent home-pen video analysis.

Table 1. Error proportion (EP) for the estimates derived from the 1 minute, 1.5 minute and 2-minute interval datasets as compared to the 30-second activity budget estimates, derived from 6hrs of footage of 7 dogs. An EP of 10% or less change from the 30-second estimate was considered an acceptable variance (Hämäläinen et al., 2016).

Behaviour	30s Estimate	EP 1min	EP 1.5min	EP 2min
Observing	39.57%	1.09%	1.09%	-2.53%
Eyes Out Of Sight	15.57%	-2.76%	0.00%	-2.76%
Lying down head or ears mobile	8.43%	-1.66%	1.66%	-15.30%
Walking	8.14%	1.84%	-5.28%	17.57%
None of the above	6.57%	-2.13%	6.54%	8.68%
Sleeping	5.86%	4.78%	4.78%	12.12%
Awake but motionless (ABM)	5.86%	2.39%	-7.34%	7.34%
Interacting with object	3.00%	-4.67%	0.00%	19.00%
Eating	1.57%	0.00%	8.92%	8.92%
Sniffing	1.57%	0.00%	18.47%	8.92%
Grooming	0.57%	-24.56%	24.56%	-24.56%
Pacing	0.57%	-24.56%	0.00%	0.00%
Abnormal behaviour	0.29%	0.00%	0.00%	48.28%
Jumping	0.14%	107.14%	107.14%	-100.00%
Urinating-excreting	0.14%	0.00%	107.14%	107.14%

Home-pen behaviour and Kong ™ preliminary results

Overall, dogs spent the longest time 'observing' (median 41.5% scans), walking (14.5%), lying down head or ears mobile (9.9%) and lying down with eyes obscured (8.0% scans) (Table S1). Crucially, the dogs did display the behaviour we hypothesised to reflect a depression-like condition [being awake but motionless 'ABM'], for a median time of 2.5% of the scans (1st quartile 1.6, 3rd quartile 4.6%) with clear variation between individual dogs (from 0 to 16.1% of scans). The initial univariate analyses used to compare the Kong variables to the activity budgets revelaed neither shelter nor age of the dog to have any association with ABM (shelter, B=-0.42, t=-0.27, p=0.788; age, B=0.73, t=1.33, p=0.203). However, sex was trending towards significance for associations with ABM, with females in this state for more scans than males ($\overline{X}_{\text{males (n = 9)}}$ = 2.44 ± 1.72 SD, 0.62-6.04, $\overline{X}_{\text{females (n = 9)}}$ = 6.31 ± 6.06 SD, 0.62-16.67, linear regression: B=3.89, t=1.85, p 0.083).

Latency to interact with the Kong™ showed no variability, with all dogs except one interacting (approaching and sniffing/licking) with it as soon as it touched the floor (less than 1 second, and just 2.5 seconds for 1 dog). For this reason, only two measures from the Kong™ Test were considered in analysis: Kong™ Time (median time 10.7sec, Q1 5.2, Q3 12.5, from 0.4 to 15.3) and Kong™ Returns (median number 3.0, Q1 2.8, Q3 6, from 1 to 12).

Time spent interacting with the Kong™ (Kong™ Time) was not associated with ABM (B=-0.23, t=-0.82, p=0.423), whilst the number of times the dog returned to interact with the Kong™ was trending towards significance (Kong™ Returns: B=-0.71, t=-1.73, p=0.103), with dogs spending greater time ABM tending to return less to the Kong™. Due to the trend towards sex being associated with ABM (p<0.1), a final multivariate model was tested, using a backwards elimination procedure, including sex and KongTM Returns. One multivariate model could be formed containing sex and KongTM Returns, both of which were significant to p<0.05 and explained a combined 37% of ABM variance (model R2 = 0.37, F = 4.47, p = 0.030).

<u>Note on refining Kong™ measures for the main study</u>: we excluded *Kong™ Latency* as this measure did not show any variability and would have, at least in theory, be influence by the original location of the dog in the kennel at the time the Kong ™ was placed on the floor (an aspect we did not think of originally). We also replaced *Kong™ Returns* by the number of bouts

and the duration of each bout based on ceasing physical contact with the Kong and ceased to chew food retrieved from the Kong™ (see main paper), as these aspects have proved to be easier to extract from the footage (*i.e.* less ambiguous) than the moving >2paces away criterion.

Sample size calculation for the main study

Sample size calculations were conducted using an online tool (www2.ccrb.cuhk.edu.hk/stat/epistudies/reg1.htm) according to methods outlined for linear regression power and sample size calculations (Dupont & Plummer, 1998). Calculations were based upon α=0.05 and β=0.2 and utilised the results of univariate linear regressions on the pilot data. Required sample sizes for the associations between ABM and the Kong™ measures and sex were: 42 for interaction bouts; 117 for total interaction time and 38 for sex differences. Whilst a sample size of 117 would be unfeasible, it was considered that a sample size of at least 42 dogs would be adequate to fully evaluate potential associations between the majority of measures for anhedonia and time spent ABM.

Dupont, W. D., & Plummer, W. D. (1998). Power and sample size calculations for studies involving linear regression. *Controlled Clinical Trials*, *19*(6), 589–601. http://doi.org/10.1016/S0197-2456(98)00037-3

Hämäläinen, W., Ruuska, S., Kokkonen, T., Orkola, S., & Mononen, J. (2016). Measuring behaviour accurately with instantaneous sampling : A new tool for selecting appropriate sampling intervals. *Applied Animal Behaviour Science*, *180*, 166–173. http://doi.org/10.1016/j.applanim.2016.04.006

Supplementary Tables

Table S1. Median percentage of scans, first (Q1) and third (Q3) quartiles and minimum and maximum values for behaviour based upon scans using 1.5 minute (n=18). The behaviour are ordered from the longest to the shortest median times spent displaying them, and the behaviour we hypothesise to specifically reflect depression is highlighted in bold.

Behaviour	Median	Q1	Q3	Min	Max
Observing	41.5%	36.3%	49.5%	11.7%	57.4%
Walking	14.5%	5.3%	23.6%	4.6%	38.9%
Lying down head or ears mobile	9.9%	5.1%	13.3%	0.0%	28.4%
Eyes out of sight (could be ABM or sleeping)	8.0%	3.5%	22.4%	0.0%	23.5%
None of the above	5.0%	2.3%	6.9%	1.9%	10.4%
**Awake but motionless (ABM)**	2.5%	1.6%	4.6%	0.6%	16.1%
Interacting with person	0.9%	0.0%	3.6%	0.0%	6.8%
Sniffing	0.8%	0.5%	2.2%	0.0%	3.1%
Barking	0.6%	0.0%	5.2%	0.0%	9.3%
Eating	0.6%	0.0%	1.1%	0.0%	8.3%
Grooming	0.6%	0.2%	1.4%	0.0%	4.9%
Interacting with object	0.6%	0.3%	1.8%	0.0%	2.5%
Abnormal behaviour	0.0%	0.0%	0.9%	0.0%	11.1%
Drinking	0.0%	0.0%	0.0%	0.0%	0.6%
Howl	0.0%	0.0%	0.0%	0.0%	0.4%
Jumping	0.0%	0.0%	2.3%	0.0%	6.2%
Sleeping	0.0%	0.0%	2.5%	0.0%	12.1%
Urinating-excreting	0.0%	0.0%	0.5%	0.0%	0.8%

Whining	0.0%	0.0%	0.0%	0.0%	0.6%

Table S2. PCA component loading table for QBA scores overlaid with Kong test variables and home-pen awake but motionless (ABM) behaviour. Highlighted in bold are the loadings above 0.5, which would be considered to be the most salient for those components. Although the loadings at the negative end of component 2 are weak (>0.3), they still represent the negative aspect for that component and did not load at all on component 1.

	Component 1	Component 2
Stressed	**0,86**	-0,20
Anxious	**0,85**	-0,25
Nervous	**0,84**	-0,10
Alert	**0,72**	0,15
Wary	**0,71**	-0,15
Comfortable	**-0,68**	0,31
Reactive	**0,64**	0,46
Fearful	**0,62**	0,05
Relaxed	**-0,58**	0,21
Excited	**0,51**	**0,50**
Interested	0,33	**0,75**
Curious	0,36	**0,71**
Explorative	-0,14	**0,70**
Playful	-0,12	0,47
Kong_Bout_N	-0,09	0,39
Kong_Prop_Time	0,02	0,37
Kong_Av_Bout_Time	0,07	-0,34
Depressed	0,07	-0,33
Home_Pen_ABM	0,07	-0,33
Bored	0,08	-0,33
Hesitant	0,00	0,03

Table S3. Kendall's tau-b correlations comparing all potential confounding variables. Three variables (female, weight and working/herding/sporting breed group) were significantly associated with each other so were always included together in any multi-variate models.

		Female	Neutered	Weeks in shelter	Age	Weight (kg)	Working/herding/sporting
Female	Rho	1	-0.06	-0.08	0.04	**-0.42****	**-0.39***
	p	.	0.671	0.525	0.76	**0.002**	**0.01**
	N		45	44	41	38	45
Neutered	Rho		1	0.05	0.07	-0.01	0.09
	p		.	0.678	0.621	0.929	0.537
	N			44	41	38	45
Weeks in shelter	Rho			1	0.10	0.05	-0.11
	p			.	0.406	0.666	0.38
	N				40	37	44
Age	Rho				1	-0.02	0.05
	p				.	0.904	0.731
	N					34	41
Weight (kg)	Rho					1	**0.57*****
	p					.	**<0.001**
	N						38

Table S4. Statistics from multi-variate general linear regressions comparing variables from the Kong™ test to time spent *awake but motionless* in the home kennel (logarithmically transformed) alongside potential confounding factors. *% Kong Time* = total time the dog spent interacting with the food toy expressed as a proportion of the total test duration. *Kong bouts (n)*: number of bouts of interaction with the Kong™. *Average bout length*: average duration of bouts of interaction with the Kong™. *% Kong Eaten*: percentage of the food mix eaten by the end of the 30 minutes test. Due to significant association between the factors 'female', 'weight(kg)' and 'working/herding/sporting' breed group, these factors were always entered together in each model and multi-collinearity was ruled out using VIF statistics which were all acceptable between 1.0 - 2.0.

**Variable**		**F**	**P**	**R^2**	**N**
% Kong Time	Alone	0.68	0.414	1.6%	43
	+ Fe, kg, breed grp	0.38	0.818	4.7%	38
	+ weeks in shelter	1.74	0.188	8.2%	42
	+ age	0.48	0.622	2.5%	40
	+ neuter status	1.48	0.239	6.9%	43
Kong Bouts (n)	Alone	0.05	0.829	0.1%	43
	+ Fe, kg, breed grp	0.40	0.806	4.9%	38
	+ weeks in shelter	1.55	0.225	7.4%	42
	+ age	0.06	0.944	0.3%	40
	+ neutered	0.76	0.474	3.7%	43
Average bout length	Alone	2.93	0.095	6.7%	43
	+ Fe, kg, breed grp	0.39	0.813	4.8%	38
	**+ weeks in shelter**	**3.66**	**0.035**	**15.8%**	**42**
	+ age	1.34	0.276	6.7%	40
	+ neutered	1.88	0.166	8.6%	43
% Kong Eaten	Alone	0.51	0.480	1.2%	43
	+ Fe, kg, breed grp	0.66	0.623	7.9%	38
	+ weeks in shelter	1.78	0.182	8.4%	42
	+ age	0.34	0.714	1.8%	40
	+ neutered	0.75	0.479	3.6%	43
